# Effects of Serum Estradiol on Proprotein Convertase Subtilisin/Kexin Type 9 Levels and Lipid Profiles in Women Undergoing In Vitro Fertilization

**DOI:** 10.3390/jcdd11010025

**Published:** 2024-01-15

**Authors:** Anna Papanikolaou, Georgia Anastasiou, Fotios Barkas, Constantinos Tellis, Konstantinos Zikopoulos, Evangelos Liberopoulos

**Affiliations:** 1 Department of Obstetrics and Gynaecology, Faculty of Medicine, University of Ioannina, 45110 Ioannina, Greece; annapapanikolaou90@gmail.com; 2 Department of Internal Medicine, Faculty of Medicine, School of Health Sciences, University of Ioannina, 45500 Ioannina, Greece; anastgeorgia@hotmail.com (G.A.); f.barkas@uoi.gr (F.B.); 3 Imperial Centre for Cardiovascular Disease Prevention, Department of Public Health and Primary Care, Faculty of Medicine, Imperial College London, Exhibition Rd, South Kensington, London SW7 2BX, UK; 4 Atherothrombosis Research Centre/Laboratory of Biochemistry, Department of Chemistry, University of Ioannina, 45110 Ioannina, Greece; ktellis@uoi.gr; 5 Genetics and IVF Unit, Department of Obstetrics and Gynaecology, Faculty of Medicine, University of Ioannina, 45110 Ioannina, Greece; kostaszikopoulos@hotmail.com; 6 1st Propedeutic Department of Medicine, School of Medicine, National and Kapodistrιan University of Athens, 11527 Athens, Greece

**Keywords:** estrogens, proprotein convertase subtilisin/kexin type 9, lipoprotein, low-density lipoprotein cholesterol, cholesterol, lipids, in vitro fertilization

## Abstract

Background: The mechanisms underlying the impact of estradiol (E2) on low-density lipoprotein cholesterol (LDL-C) levels are not completely understood, although a role for proprotein convertase subtilisin/kexin type 9 (PCSK9) has been proposed. We aimed to investigate the association between levels of E2, PCSK9, and lipid parameters in premenopausal women undergoing in vitro fertilization (IVF). Methods: Healthy women undergoing IVF in the Department of Obstetrics and Gynecology of the University General Hospital of Ioannina were recruited. Their levels of E2, PCSK9, total cholesterol (TC), high-density lipoprotein cholesterol (HDL-C), LDL-C, and triglycerides (TGs) were measured 10 days after ovarian depression (E2min) and 7 days after ovarian stimulation (E2max). Results: We included 34 consecutive women of median age 38 (interquartile range 26–46) years who underwent a full IVF cycle. As expected, E2 levels increased by 329.6% from E2min to E2max (108 [47–346] to 464 [241–2471] pg/mL, *p* < 0.05). During the same time, serum PCSK9 levels decreased by 30.8% (245 ± 80 to 170 ± 64 ng/mL, *p* < 0.05). TC, LDL-C, and TGs decreased by 0.4%, 3.8%, and 2.2%, respectively, while HDL-C levels increased by 5.3% (all *p* = NS). Conclusions: The rise in endogenous E2 during an IVF cycle was related with a significant decline in serum PCSK9 levels, but no significant change in plasma lipids during a 7-day period.

## 1. Introduction

Premenopausal women have lower-plasma low-density lipoprotein cholesterol (LDL-C) compared with postmenopausal ones and men [1]. LDL particles are predominantly removed from the bloodstream through hepatic uptake facilitated by the LDL receptor (LDLR) [2]. The expression of the LDLR is regulated at the transcriptional level by the sterol regulatory element-binding protein 2 (SREBP-2), and at the post-translational level primarily by proprotein convertase subtilisin/kexin-type 9 (PCSK9), a regulator that prompts the degradation of the LDLR [2,3]. Specifically, SREBP-2, a member of the nuclear transcription factor family, binds to the sterol regulator in lipid synthetic enzyme gene promoters, activating transcription and gene transcription in the cholesterol biosynthesis pathway [3]. The SREBP-2 gene has been implicated in various metabolic disorders, including obesity and metabolic syndrome [3]. Circulating PCSK9 attaches to the LDLR, resulting in lysosomal degradation within the cells [4]. This process subsequently results in decreased LDLR expression on the cell membrane, decreased LDL catabolism, and elevated plasma LDL-C levels [4]. 

Estrogens increase LDLR expression through estrogen receptor a (ERa), which is known to have a genomic effect and inhibits the negative feedback regulation of cholesterol synthesis via the SREBP-2 pathway [5,6,7,8,9,10]. Furthermore, activation of the G protein-coupled estrogen receptor (GPER) by estrogen upregulates LDLR expression and downregulates PCSK9 via a non-genomic, post-transcriptional effect [11]. In HepG2 cells expressing this receptor, the activation of GPER by its agonist results in a decrease in both PCSK9 mRNA and protein levels [12]. Consequently, LDLR protein expression on the cellular membrane is increased [12]. Studies in both rats and humans have demonstrated that elevated levels of endogenous estrogens lead to a reduction in serum PCSK9 [13,14,15]. This suggests that estrogens also increase the number of LDLRs in hepatic cells through a post-transcriptional mechanism [13,14,15]. Indeed, PCSK9 levels are lower in women than in men, and higher in postmenopausal vs. premenopausal women [16]. In experimental studies, high-dose ethinylestradiol induced a 4-fold increased LDLR gene expression in conjunction with a 50% inhibition of hepatic PCSK9 gene expression [14].

We aimed to investigate the short-term effect of increased endogenous estradiol (E2) levels on serum PCSK9 and lipid levels. To this end, we established a human model to determine the relationship between changes in E2 levels and those in PCSK9 and lipids, following ovarian suppression and stimulation during an in vitro fertilization (IVF) cycle. 

## 2. Materials and Methods

### 2.1. Study Design

This was a prospective study of women who were scheduled for IVF at the Department of Assisted Reproduction at the University General Hospital of Ioannina, Ioannina, Greece.

To be included in this study, women had to have been healthy, non-smokers, not under any other medication, aged 18–48 years, and with a BMI of 18.5–29.0 kg/m^2^. This should have been their first IVF cycle after a 12-month period of regular unprotected sexual intercourse which failed to achieve pregnancy. Women below 18 or over 48 years, smokers, on any kind of medication, obese or underweight, or with previous IVF attempts or previous pregnancies, regardless of the outcome (birth, abortion, or miscarriage), were excluded.

**Figure 1:**
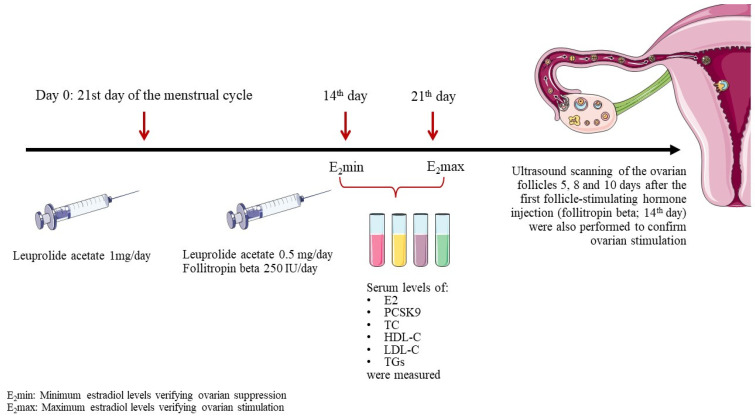
Study design. Abbreviations: E2, estradiol; PCSK9, proprotein convertase subtilisin/kexin type 9; TC, total cholesterol; HDL-C, high-density lipoprotein cholesterol; LDL-C, low-density lipoprotein cholesterol; TGs, triglycerides.

To induce controlled ovarian stimulation, endogenous estrogens were first suppressed using the gonadotropin-releasing hormone agonist leuprolide acetate. Leuprolide acetate was administered subcutaneously at a dosage of 1 mg/day starting on the 21st day of the menstrual cycle. Fourteen days later, an E2 measurement (E2min) was carried out to verify suppression. Subsequently, the leuprolide acetate dose was decreased to 0.5 mg/day and ovarian stimulation was initiated via subcutaneous administration of the recombinant human follicle-stimulating hormone follitropin beta, at a dosage of 250 IU/day. The response was measured with an E2 measurement 7 days later (E2max) and via ultrasound scanning of the ovarian follicles 5, 8, and 10 days after the first follicle-stimulating hormone injection (Figure 1).

Serum levels of E2, PCSK9, total cholesterol (TC), high-density lipoprotein cholesterol (HDL-C), LDL-C, and triglycerides (TGs) were measured at two time points: E2min and E2max.

All subjects gave informed consent to participate, and the study protocol was approved by the Ethics Committee of University General Hospital of Ioannina.

### 2.2. Collection and Analysis of Blood Samples

Blood samples were collected in EDTA tubes and centrifuged for 15 min at 1000× *g* within 30 min of collection to separate the plasma, which was stored at −80 °C. All laboratory determinations were carried out after a 12 h overnight fast.

Free E2 was measured using routine clinical assays (Estradiol Reagent Kit, Alinity, Abbott) (detection limit, 150 pg/mL). ApoB-depleted plasma was isolated after the sedimentation of all ApoB-containing lipoproteins with dextran sulfate–magnesium chloride, as previously described, and stored at −80 °C until analysis. TCs and HDL-C were determined enzymatically in plasma and ApoB-depleted plasma, respectively, using a commercially available kit (Human Diagnostics, reference number 10028; Wiesbaden, Germany) according to the manufacturer’s instructions with slight modifications. Briefly, 5 μL total plasma samples or 10 μL ApoB-depleted plasma, and a standard of varying volumes (0–20 μL of 200 mg/dL cholesterol standard reagent), were added into each well of the microplate (Costar 3370, Corning, NY, USA) and mixed with working reagent (cholesterol enzyme and substrates mixture) in a 300 μL final volume. The reaction was performed for 10 min at 37 °C. The optical density of each well was measured at 510 nm using a microplate reader (Tecan infinite 200 PRO). Total plasma and ApoB-depleted plasma (HDL-rich content) cholesterol of the samples were calculated (mg/dL) using a standard curve. Plasma TGs were determined using a commercially available kit (Human Diagnostics, reference number 10724; Wiesbaden, Germany) according to the manufacturer’s instructions with slight modifications. Briefly, 5 μL total plasma samples, and a standard of varying volumes (0–20 μL of 200 mg/dL TG standard reagent) were added into each well of the microplate (Costar 3370 Corning, New York, USA) and mixed with working reagent at a 300 μL final volume. The reaction was performed for 10 min at 37 °C. The optical density of each well was measured at 510 nm using a microplate reader (Tecan infinite 200 PRO). Plasma TGs (mg/dL) were calculated using a standard curve. Samples and standards were assayed in duplicates. LDL-C was calculated by using the Friedewald formula (LDL-C = TC − TGs/5 − HDL-C) (if TGs were <400 mg/dL (4.52 mmol/L)). The Human PCSK9 Quantikine ELISA kit (R&D Systems, cat. no. DPC900, Minneapolis, MN, USA) was used to measure the PCSK9, according to manufacturer’s instructions. Briefly, a monoclonal antibody specific for human PCSK9 was pre-coated on a microplate (ready to use). Fifty (50) µL of the standards and samples were pipetted into the wells and the plate was incubated for 2 h at room temperature, where any PCSK9 present is bound by the immobilized antibody. Then, each well was aspirated and washed 3 times with cleaning buffer (400 µL/well). After washing away unbound conjugates, an enzyme-linked polyclonal antibody specific for human PCSK9 was added to the wells (200 µL per well) and the plate was incubated for 2 h at room temperature. The plate was then washed to remove any unbound antibodies/enzyme reagent, and 200 µL of substrate solution was added to the wells and incubated for 30 min at room temperature. The color of the solution develops in proportion to the amount of bound PCSK9 at the initial stage. Color development was stopped (blue) by adding 50 µL of stop solution, and the intensity of the yellow color was measured using a microplate reader set at 450 nm. A standard curve was generated, with the ordinate mean absorbance for each standard on the y-axis versus the concentration on the x-axis. The data in this study can be linearized by plotting the log of human PCSK9 concentrations against the log of optical density (OD), and the best fit can be determined via regression analysis. The minimum detectable dose (MDD) of human PCSK9 ranges from 0.030 to 0.219 ng/mL, and the mean MDD is 0.096 ng/mL. The intra- and inter-test CVs are 6.1 and 5.9%, respectively.

### 2.3. Statistical Analysis

Statistical analysis was performed using the Statistical Package for Social Sciences (SPSS) 21.0 software (SPSS Statistics for Windows, Version 28.0. Armonk, New York, NY, USA: IBM Corp.). Continuous numeric variables are expressed as mean ± standard deviation (SD) or median (interquartile: IQR) if Gaussian or non-Gaussian distributed, respectively. Correlations between parameters were evaluated using Pearson’s and Spearman’s correlation coefficients. A paired-sample *t*-test (parametric and non-parametric) was performed to investigate the change in numeric variables during follow-up. Two-tailed significance was defined as *p* < 0.05. As this is a pilot study, no official power calculations were performed.

## 3. Results

Thirty-four consecutive women, with a median age of 38 (18–48) years, were included in this study. 

Table 1 shows the participants’ baseline characteristics. 

Table 2 shows changes in the E2, PCSK9, and lipid levels between the E2min and E2max time points. As expected, E2 levels increased by almost 330% (*p* < 0.05). PCSK9 levels were significantly reduced by 30.8% between these two time points (*p* < 0.05). ΔPCSK9 was significantly correlated with ΔE2 (beta = −0.162, *p* = 0.001). TC, TG, and LDL-C levels decreased by 0.4%, 2.2%, and 3.8%, respectively (*p* = NS). HDL-C levels increased by 5.3% (*p* = NS). No significant correlations were found between changes in the E2 and TC, LDL-C, TG, and HDL-C levels. Additional analysis was performed for two age subgroups: (1) 25–36 (N = 9) and (2) 37–49 years (N = 29). In the first group, E2 levels increased by 335%, PCSK9 levels were reduced by 40.1% (Table 3), and ΔPCSK9 was significantly correlated with ΔE2 (beta = −0.212, all *p* < 0.05). In the second group, E2 levels increased by 324%, PCSK9 levels were reduced by 28.6% (Table 4), and ΔPCSK9 was significantly correlated with ΔE2 (beta = −0.193, all *p* < 0.05). ΔPCSK9 was significantly higher in the first age subgroup compared with the second (40.1% vs. 28.6%, *p* < 0.05).

## 4. Discussion

In this study, we demonstrated that short-term (7 days) elevation of endogenous E2 during an IVF cycle was associated with a significant reduction in PCSK9 levels and non-significant alterations in lipid profiles. Additionally, we showed that ΔPCSK9 was significantly higher in the younger age subgroup compared with the older.

Estrogen’s role in lipid regulation extends beyond its influence on LDL receptor (LDLR) expression through the nuclear receptor estrogen receptor a (ERa) [5,6,7,8,9,10]. It involves intricate mechanisms affecting cholesterol synthesis, transport, and metabolism. Estrogen receptors (ERs), particularly ERα, play a crucial role in modulating lipid-related gene expression. ERα complexes with transcription factors like Sp1 bind to the LDLR promoter and promote LDLR transcription, thereby increasing LDL uptake from circulation [10]. Emerging evidence highlights estrogen’s direct effect on PCSK9 transcription. Studies indicate that estrogen, notably E2, downregulates PCSK9 expression, contributing to enhanced LDLR levels [14]. This complex interplay involves G protein-coupled estrogen receptor (GPER) activation, which impedes PCSK9-mediated LDLR degradation. This dual action of estrogen—enhancing LDLR expression and reducing PCSK9—contributes to lower LDL-C levels. Specifically, the G protein-coupled estrogen receptor (GPER) conveys the impact of estrogen on LDL-C via a non-genomic, post-transcriptional effect [12,17,18]. Of interest, estrogens upregulate LDLR levels mainly through GPER activation, which inhibits PCSK9-dependent LDLR degradation in HepG2 cells [19]. In another study, Starr et al. showed that PCSK9 is necessary for post-transcriptional upregulation of LDLR by E2 in human hepatic HuH7 cells [20]. Furthermore, studies in rats and humans have found that high levels of endogenous estrogens decline serum PCSK9 levels, indicating that estrogens also increase the density of LDLRs in hepatic cells via a post-transcriptional pathway [13,14,15]. Furthermore, Persson et al. reported that high-dose treatment with ethinylestradiol decreased SREBP-2 mRNA levels by 55%, and reduced PCSK9 expression by half [14]. 

Interestingly, analysis of ovariectomized or unmodified PCSK9 knockout mice administered with E2 or a placebo showed that female mice, but not males, significantly release the soluble ectodomain of the LDLR into the bloodstream [21]. In addition, greater liver LDLR protein density was observed in male but not female PCSK9 knockout mice [21]. Ovariectomized knockout female mice administered with a placebo exhibited results similar to a typical knockout male, while those receiving E2 supplementation exhibited surface patterns resembling those of wild-type females [21]. When male mice were administrated E2, LDLR density at the hepatocyte cell surface was reduced and the soluble LDLRs in their plasma were increased in a female-like pattern [21]. Notably, a sole injection of E2 in PCSK9 knockout ovariectomized female mice resulted in a 64% reduction in surface LDLR labeling after 24 h [21]. Similarly, a sole injection of E2 in PCSK9 knockout males caused a significant decrease in LDLR labelling after 24 h. This study indicates the existence of an ERa-dependent pathway to affecting the surface LDLR density in liver cells [21].

Long-term estrogen replacement therapy in postmenopausal women demonstrates substantial lipid profile modifications. Studies report significant reductions in LDL-C and elevations in HDL-C levels following extended estrogen exposure [22,23,24,25,26]. Interestingly, one study investigated the effect of oral and transdermal estrogen replacement therapy on serum lipid parameters in 90 hysterectomized and oophorectomized women [22]. The female participants were randomized into three study groups: (1) those who received no hormones, (2) those who received transdermal estradiol patches (50 µg/day), and (3) those who received oral estrogen (0.625 mg/day) [22]. According to the study results, the oral estrogen replacement therapy significantly reduced LDL-C levels from 127.46 ± 36.93 mg/dL to 104.13 ± 34.66 mg/dL after 3 months and 99.26 ± 33.55 mg/dL after 6 months [22]. Similarly, a significant decline in LDL-C levels was observed in the female individuals treated with transdermal estrogen patches, from 129.83 ± 37.22 mg/dL to 125.63 ± 58.22 mg/dL after 3 months and 109.00 ± 36.58 mg/dL after 6 months of treatment [22]. In addition, a significant rise in HDL-C levels in both the oral and transdermal therapy study groups was seen (from 30.63 ± 6.97 mg/dL to 42.06 ± 7.72 mg/dL after 3 months in the study group receiving oral estrogen therapy, *p* < 0.01, and from 30.90 ± 6.96 mg/dL to 33.30 ± 6.67 mg/dL after 3 months, *p* < 0.05, in the study group receiving therapy with transdermal estrogen patches) [22]. In a study of 1057 postmenopausal women (n = 328 estrogen users), estrogen use was associated with lower LDL-C levels and higher HDL-C, and this correlation was observed to be dependent on both the dosage and duration of therapy [23]. 

Interestingly, some studies demonstrate a dose-dependent correlation between estrogens and lipid parameters. Specifically, one study of 1678 postmenopausal women (n = 585 current users of conjugated estrogen: 388 higher-dose users (≥1.25 mg) and 197 lower-dose users (≤0.625 mg); and n = 1093 non-users) showed that the effect of estrogen administration is associated with LDL-C, HDL-C, and TG levels (*p* < 0.05) in a dose-dependent manner, with the greatest effect observed at a dosage of 1.25 mg/day [24]. In the same line, in a prospective study of 2270 women (n = 593 contraceptive estrogen users and n = 1677 non-users), HDL-C (67 vs. 57 mg/dL) and TG levels (167 vs. 142 mg/dL) were significantly greater in estrogen users compared with non-users [25]. On the contrary, LDL-C levels were lower in the estrogen users (145 vs. 156 mg/dL) vs. the non-users [25]. Similar results were shown in a study of 55 postmenopausal women who were randomized on either 2 mg estradiol valerate (E_2_V) (intermittent treatment plan: 21 consecutive days of treatment followed by a subsequent 7-day period free from treatment) or 2 mg E_2_V combined with 1 mg cyproterone acetate (E_2_V + CPA) daily, for 6 months [26]. In this research, LDL-C levels were reduced by 9.7% and by 1.8% after 1 month, and by 8.7% and 2.1% after 6 months in the E_2_V and E2V + CPA study groups, respectively (*p* > 0.05) [26]. TC levels were reduced in both study groups and HDL-C levels were increased (*p* > 0.05) [25]. One prospective, controlled study investigated the effect of estrogen therapy in 184 women who had been amenorrheic for over 1 year without a hysterectomy or had serum follicle-stimulating hormone concentrations over 20 mlU/L [27]. The women were stratified into four study groups: group A (n = 29), a control group without medication; group B (n = 67), treated with 0.625 mg conjugated estrogen and 10 mg medroxyprogesterone acetate; group C (n = 65), treated with 0.625 mg conjugated estrogen and 5 mg medroxyprogesterone acetate; and group D (n = 23), treated with 0.625 mg conjugated estrogen alone in subjects with a prior hysterectomy [27]. According to the study’s findings, LDL-C levels were significantly declined from 131.1 ± 25.8 mg/dL, 131.3 ± 29.4 mg/dL, and 139.7 ± 27.0 mg/dL to 120.0 ± 24.7 mg/dL, 119.1 ± 28.8 mg/dL, and 127.6 ± 26.9 mg/dL in groups B (*p* < 0.001), C (*p* < 0.001), and D (*p* < 0.05), respectively, within 2 months of treatment [27]. However, increases in HDL-C levels were observed only in study group D, from 66.5 ± 15.2 mg/dL to 72.3 ± 17.3 mg/dL (*p* < 0.05) [27]. 

Of note, short-term studies like the current IVF cycle-related investigation show varying outcomes. In a previous short-term study (6-day stimulation of estrogen synthesis) of 31 women who underwent IVF, TC, LDL-C, and PCSK9 levels were significantly reduced by 14.0%, 20.2%, and 14.0%, respectively, while HDL-C levels remained unchanged [28]. In the current study, PCSK9 levels decreased by 31% (*p* < 0.05), and this decrease was significantly correlated with an increase in E2 levels (beta = −0.162, *p* = 0.001). However, TC, TGs, LDL-C, and HDL-C did not significantly change (−0.4%, −2.2%, −3.8%, and +5.3%, respectively, all *p* = NS). 

We demonstrated a significantly greater percentage reduction of PCSK9 in younger women vs. older women. The reasons for this are unclear, but may be related to the higher sensitivity of ERs in the younger women, possibly leading to an increased impact of E2 on PCSK9 [29]. 

While our study revealed a notable decrease in PCSK9 levels without significant alterations in lipid profiles, contrasting findings in other short-term studies suggest a need for further exploration. These discrepancies could arise from diverse factors like variations in the duration and method of estrogen administration. For instance, the discrepancy in E2 increase between studies might stem from distinct protocols used for ovarian stimulation. Indeed, E2 increased by 330% in our study as compared with 3720% in the Persson et al. study. This difference in E2 change between these two studies could be due to the use of a gonadotropin nasal spray in Persson’s study instead of the subcutaneous gonadotropin in our study [28]. Other potential factors for the lack of significant changes in the plasma lipids may include differences in diet and baseline lipids, as well as the genetic background. These disparities emphasize the importance of considering study design and methodologies when interpreting results.

It is worth mentioning here that men and women may respond differently to PCSK9 inhibitors. Indeed, in a recent meta-analysis of 16 clinical trials with 54,996 adults (27.5% were females), LDL-C reduction with PCSK9 inhibitors was greater in males than in females [30]. Specifically, their LDL-C levels were significantly reduced at 12 weeks (females: mean difference (MD) 62.57, 95% CI: −70.24 to −54.91, *p* < 0.001; males: MD −66.19, 95% CI: −72.03 to −60.34, *p* < 0.001) and 24 weeks (females: MD −47.52, 95% CI: −52.94 to −42.09, *p* < 0.001; males: MD −54.07, 95% CI: −59.46 to −48.68, *p* < 0.001) [30]. Notably, the study demonstrated that sex-specific differences were significant at both 12 weeks (males vs. females: MD −4.55, 95% CI: −7.34 to −1.75, *p* < 0.01) and 24 weeks of treatment (males vs. females: MD −7.11, 95% CI: −9.99 to −4.23, *p* < 0.001) [30]. The underlying mechanisms behind these sex-specific differences in LDL-C reduction are not well understood, but an E2-associated reduction in PCSK9 levels, as shown in this study, may affect the effectiveness of PCSK9 inhibition.

Recognizing the limitations of existing research in this field—such as small sample sizes and short study durations—evidences the need for larger, longer-term investigations with control groups. Addressing these limitations could unravel more conclusive associations between short-term estrogen elevation, PCSK9 modulation, and lipid profile changes. Further research could elucidate the clinical implications and therapeutic potentials of managing lipid-related disorders.

## 5. Conclusions

We have shown that a short-term increase in E2 during an IVF cycle is associated with a decrease in serum PCSK9 levels, but no significant alteration in plasma lipids. The clinical implications of these findings are uncertain.

## Figures and Tables

**Table 1:** The studied women’s baseline characteristics (*n* = 34).

Age (years)	38 (18–48)
Weight (kg)	64 (55–78)
BMI (kg/m^2^)	22.8 (19.1–26.7)
Smokers (%)	0

Abbreviations: BMI, Body Mass Index. Values are expressed as the median (IQR), unless percentages are shown.

**Table 2:** E2, PCSK9, and serum lipid levels of the study participants (n = 34) at ovarian depression (E2min) and ovarian stimulation (E2max).

	E2min	E2max	Change, %
E2, pg/mL	108 (47–346)	464 (241–2471)	+329.6 *
ΤC, mg/dL	164 ± 44	163 ± 42	−0.4
TGs, mg/dL	69 ± 36	67 ± 24	−2.2
LDL-C, mg/dL	108 ± 43	103 ± 42	−3.8
HDL-C, mg/dL	42 ±10	45 ± 14	+5.3
PCSK9, ng/mL	245 ± 80	170 ± 64	−30.8 *

Abbreviations: E2; estradiol, TC; total cholesterol, TGs; triglycerides, LDL-C; low-density cholesterol, HDL-C; high-density cholesterol, PCSK9; proprotein convertase subtilisin/kexin type 9. Values are expressed as the median (interquartile range: IQR) or mean ± standard deviation (SD), * *p* < 0.05 for comparison versus the Emingroup.

**Table 3:** E2, PCSK9, and serum lipid levels of the 25–36 year age subgroup of women (n = 9) at ovarian depression (E2min) and ovarian stimulation (E2max).

	E2min	E2max	Change, %
E2, pg/mL	101 (56–327)	439 (208–794)	+334.6% *
ΤC, mg/dL	155 ± 40	155 ± 36	0%
TGs, mg/dL	83 ± 34	79 ± 24	−54.9%
LDL-C, mg/dL	102 ± 39	100 ± 34	−1.7%
HDL-C, mg/dL	37 ± 5	39 ± 7	+5.7%
PCSK9, ng/mL	239 ± 60	143 ± 25	−40.1% *^

Abbreviations: E2; estradiol, TC; total cholesterol, TGs; triglycerides, LDL-C; low-density cholesterol, HDL-C; high-density cholesterol, PCSK9; proprotein convertase subtilisin/kexin type 9. Values are expressed as the median (interquartile range: IQR) or mean ± standard deviation (SD), * *p* < 0.05 for comparison versus the E2min group, and ^ *p* < 0.05 for comparison versus the second age subgroup.

**Table 4:** E2, PCSK9, and serum lipid levels of the 37–49 year age subgroup of women (n = 29) at ovarian depression (E2min) and ovarian stimulation (E2max).

	E2min	E2max	Change, %
E2, pg/mL	115 (47–346)	488 (265–2471)	+324.3% *
ΤC, mg/dL	169 ± 47	166 ± 45	−2.1%
TGs, mg/dL	64 ± 36	63 ± 23	−0.7%
LDL-C, mg/dL	112 ± 46	106 ± 45	−5.5%
HDL-C, mg/dL	45 ± 10	47 ± 14	+4.9%
PCSK9, ng/mL	248 ± 85	177 ± 71	−28.6% *

Abbreviations: E2; estradiol, TC; total cholesterol, TGs; triglycerides, LDL-C; low-density cholesterol, HDL-C; high-density cholesterol, PCSK9; proprotein convertase subtilisin/kexin type 9. Values are expressed as the median (interquartile range: IQR) or mean ± standard deviation (SD), * *p* < 0.05 for comparison versus the E2min group.

## Data Availability

Data are unavailable due to privacy and local ethical restrictions.
